# Pseudomonas aeruginosa FpvB Is a High-Affinity Transporter for Xenosiderophores Ferrichrome and Ferrioxamine B

**DOI:** 10.1128/mbio.03149-22

**Published:** 2022-12-12

**Authors:** Derek C. K. Chan, Lori L. Burrows

**Affiliations:** a David Braley Center for Antibiotic Discovery, Michael G. DeGroote Institute for Infectious Disease Research, Department of Biochemistry and Biomedical Sciences, McMaster University, Hamilton, Ontario, Canada; Geisel School of Medicine at Dartmouth

**Keywords:** *Pseudomonas aeruginosa*, TonB-dependent transporter, antibiotic uptake, iron acquisition, pyoverdine, siderophores

## Abstract

Iron is essential for many biological functions in bacteria, but its poor solubility is a limiting factor for growth. Bacteria produce siderophores, soluble natural products that bind iron with high affinity, to overcome this challenge. Siderophore-iron complexes return to the cell through specific outer membrane transporters. The opportunistic pathogen Pseudomonas aeruginosa makes multiple transporters that recognize its own siderophores, pyoverdine and pyochelin, and xenosiderophores produced by other bacteria or fungi, which gives it a competitive advantage. Some antibiotics exploit these transporters to bypass the membrane to reach their intracellular targets—including the thiopeptide antibiotic, thiostrepton (TS), which uses the pyoverdine transporters FpvA and FpvB to cross the outer membrane. Here, we assessed TS susceptibility in the presence of various siderophores and discovered that ferrichrome and ferrioxamine B antagonized TS uptake via FpvB. Unexpectedly, we found that FpvB transports ferrichrome and ferrioxamine B with higher affinity than pyoverdine. Site-directed mutagenesis of FpvB coupled with competitive growth inhibition and affinity label quenching studies suggested that the siderophores and antibiotic share a binding site in an aromatic pocket formed by the plug and barrel domains but have differences in their binding mechanism and molecular determinants for uptake. This work describes an alternative uptake pathway for ferrichrome and ferrioxamine B in P. aeruginosa and emphasizes the promiscuity of siderophore transporters, with implications for Gram-negative antibiotic development via the Trojan horse approach.

## INTRODUCTION

Iron is an essential micronutrient for bacteria but has poor aqueous solubility at neutral pH and, consequently, low bioavailability (1, 2). At sites of infection, bacteria also compete with host-defense proteins that sequester iron. To overcome these limitations, Gram-negative bacteria secrete siderophores, small molecules with high affinity for iron. Once outside the cell, siderophores scavenge iron and return through specific outer membrane transporters on the cell surface (3). The architecture of siderophore transporters is conserved, consisting of a 22-stranded beta-barrel with a plug domain that occludes the lumen to prevent passive diffusion (3). The extracellular loops of the transporters are important for siderophore recognition and uptake. The periplasmic N terminus contains a short motif known as the TonB box (3–5), which interacts with the inner membrane protein TonB. Together with the inner membrane proteins ExbB-ExbD, TonB harnesses the proton motive force to actively transport ligands through the transporters via a mechanism that remains incompletely understood (2, 3). Although TonB-dependent transporters (TBDTs) are considered ligand specific, they can be exploited by antimicrobial compounds, bacteriophages, and bacteriocins for uptake, making them of interest for drug delivery across the outer membrane of Gram negatives (6–14).

**FIG 1:**
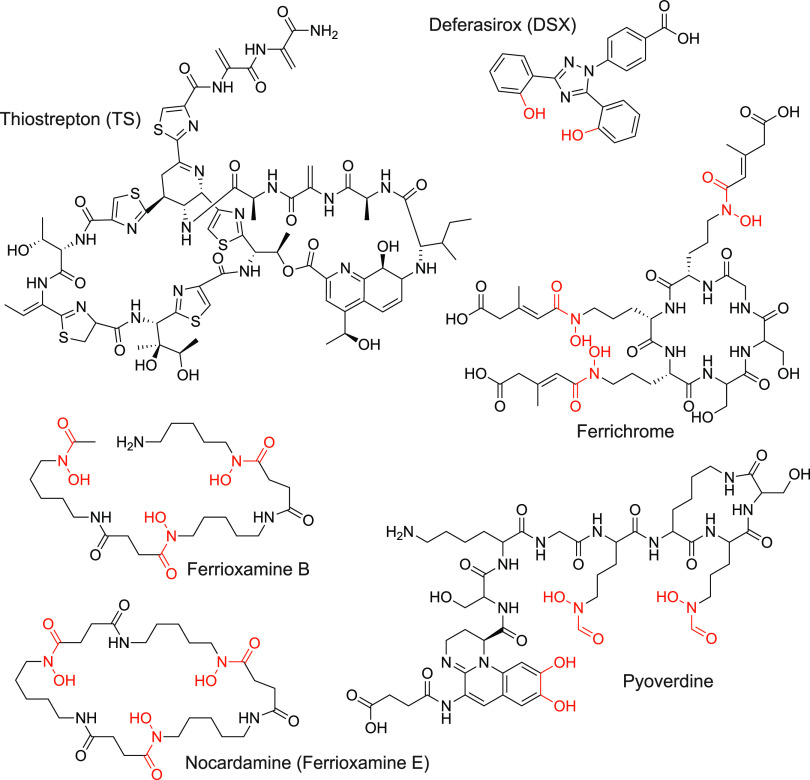
Structures of compounds used in this study. Iron chelating groups are highlighted in red.

The opportunistic bacterial pathogen, Pseudomonas aeruginosa encodes ~35 predicted TBDTs for different ligands, including siderophores, cobalamin, and other metal complexes (2, 4, 8, 15–17). P. aeruginosa makes two main siderophores, pyoverdine and pyochelin, which are taken up via FpvA and FpvB (pyoverdine) and FptA (pyochelin), respectively (18–22) (Fig. 1). Pyoverdine has a higher affinity for iron than pyochelin (23, 24) and has roles in tolerance to antibiotics, biofilm formation, and virulence factor production (25, 26). P. aeruginosa can also take up siderophores produced by other microorganisms, including ferrioxamine E and B (produced by *Streptomyces* spp.) and ferrichrome (produced by fungal species) via the FoxA and FiuA TBDTs, respectively (2, 17, 27) (Fig. 1).

Understanding the range of ligands that can be taken up by TBDTs is important, as there is growing interest in designing novel antibiotics that can exploit these transporters for uptake. However, our understanding of the substrate range for individual TBDTs is lacking. Even for those with known ligands, there may be substrates that have yet to be discovered. For example, ferrioxamine E exclusively uses FoxA to enter P. aeruginosa (27). However, after *foxA* or *fiuA* are deleted, the bacteria still grow in iron-limited media when supplied with ferrioxamine B or ferrichrome, respectively, suggesting that these siderophores can also be recognized by other transporters (27).

Previously, we showed that the large-thiopeptide antibiotics thiostrepton (TS) and thiocillin use TBDTs to enter P. aeruginosa to access their cytoplasmic target, the ribosome. TS exploits the pyoverdine transporters, FpvA and FpvB, while thiocillin uses the ferrioxamine transporter, FoxA (Fig. 1) (13, 14, 28). Here, we further characterized the interaction of TS with the pyoverdine transporters. We discovered that the secondary transporter FpvB has high affinity for ferrichrome and ferrioxamine B but is a poor pyoverdine transporter. FpvB is a promiscuous transporter, as it can recognize structurally distinct ligands using different binding modes. Overall, this work expands our understanding of TBDT function and fills in the missing details of ferrichrome and ferrioxamine B uptake in P. aeruginosa.

## RESULTS

### Exogenous pyoverdine poorly rescues iron-restricted growth of a PA14 Δ*fpvA* mutant.

Previously, we showed that TS synergized with the FDA-approved iron chelator deferasirox (DSX) against PA14 and that susceptibility required the FpvA and FpvB pyoverdine transporters (Fig. 1) (13). DSX lacks antimicrobial activity against wild-type (WT) cells since they produce pyoverdine, which competes with DSX for iron. Therefore, WT PA14, a Δ*fpvA* mutant, and a Δ*fpvB* mutant are each expected to be susceptible to TS and grow in the presence of DSX since they still encode functional pyoverdine transporters. A Δ*fpvA* Δ*fpvB* mutant is resistant to TS and inhibited by DSX (13, 14).

**FIG 2:**
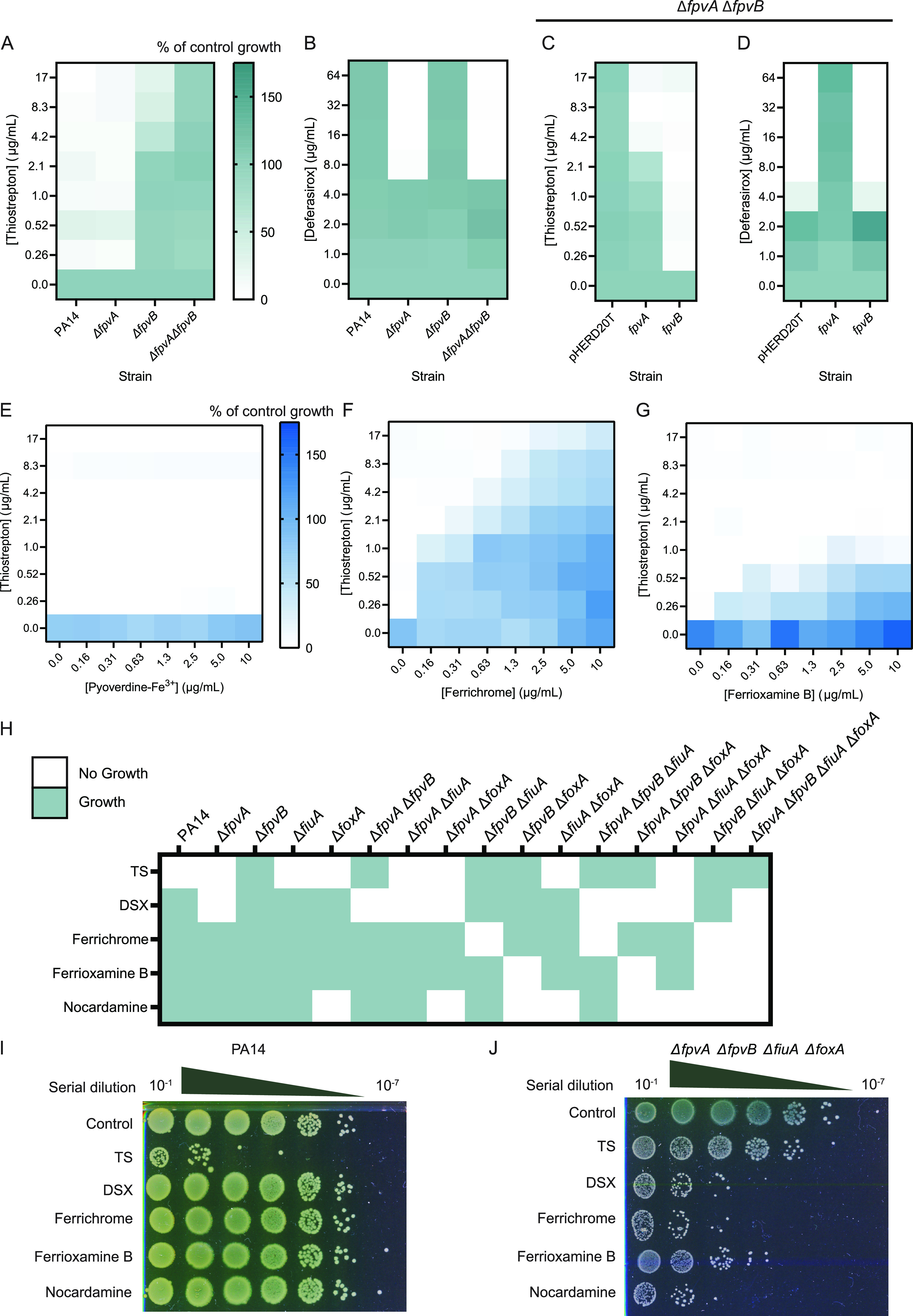
FpvB is required for growth with ferrichrome and ferrioxamine B in the absence of FiuA and FoxA. PA14, Δ*fpvA*, Δ*fpvB*, and Δ*fpvA* Δ*fpvB* strains treated with TS (A) and DSX (B) in 10:90. Green indicates growth, and white indicates no growth. Growth is expressed as percent of control. The PA14 Δ*fpvA* Δ*fpvB* strain complemented with pHERD20T, *fpvA*, or *fpvB* treated with TS (C) and DSX (D) in 10:90. Checkerboard assays of the PA14 Δ*fpvA* Δ*fpvB* pHERD20T-*fpvB* strain treated with TS and pyoverdine-Fe^3+^ (E), ferrichrome (F), and ferrioxamine B (G) in 10:90 + 2% arabinose. All experiments are averaged from three independent biological replicates. Blue indicates growth and white indicates no growth. (H) Summary of growth phenotypes of WT PA14 and combinations of single, double, triple, and quadruple deletion mutants in *fpvA*, *fpvB*, *fiuA*, and *foxA.* Strains were treated with 17 μg/mL TS, 64 μg/mL DSX, 10 μg/mL ferrichrome, 10 μg/mL ferrioxamine B, or 10 μg/mL ferrioxamine E in 10:90. Green indicates growth, whereas no growth is indicated in white. Serial 10-fold dilutions of PA14 (I) and the quadruple mutant (J) treated with 17 μg/mL TS, 64 μg/mL DSX, 10 μg/mL ferrichrome, 10 μg/mL ferrioxamine B, or 10 μg/mL ferrioxamine E in 10:90 spotted onto LB 1.5% agar plates and grown overnight at 37°C. Representative plates are shown.

We first confirmed these phenotypes via MIC assays. The WT and Δ*fpvA* mutant were susceptible to TS in iron-limited medium (Fig. 2A). The Δ*fpvB* mutant was resistant to TS, although its growth was reduced at the maximum soluble TS concentration of 17 μg/mL. The Δ*fpvA* Δ*fpvB* mutant was resistant to TS with no observable decrease in growth. The MIC of DSX against the Δ*fpvA* Δ*fpvB* mutant was 8 μg/mL (Fig. 2B), but unexpectedly, DSX also inhibited the growth of the Δ*fpvA* mutant with the same MIC. This result was surprising, since the Δ*fpvA* mutant has WT susceptibility to TS, suggesting that FpvB is expressed in that background. To confirm that FpvB was expressed in the Δ*fpvA* mutant, we tagged FpvB chromosomally with a C-terminal FLAG tag in both WT and the Δ*fpvA* mutant and blotted for expression. FpvB was detected in the tagged WT and Δ*fpvA* mutant but not in the untagged strains (see Fig. S1A in the supplemental material). FpvA is also expressed in WT cells (Fig. S1B). As a loading control, we monitored expression of PilF, an outer membrane lipoprotein required for multimerization and localization of the P. aeruginosa type IV pilus secretin (29). Taken together, these results suggested that the role of FpvB in pyoverdine transport needed to be revisited.

10.1128/mbio.03149-22.1 FIG S1 (A) FpvB is expressed in WT PA14 and the Δ*fpvA* mutant grown in 10:90. PilF was used as a loading control for outer membrane proteins. (B) FpvA is expressed from WT PA14 grown in 10:90. No band was seen for untagged FpvA in WT PA14. PilF was used as a loading control for outer membrane proteins. (C) Growth curves of PA14 and mutants with DSX and different siderophores in 10:90. PA14, Δ*fpvA*, Δ*fpvB*, Δ*fpvA* Δ*fpvB*, and Δ*pvdA* Δ*pchA* strain growth. Blue indicates control, purple indicates treatment with 64 μg/mL DSX, yellow indicates treatment with 10 μg/mL pyoverdine (PA14), and red indicates treatment with 64 μg/mL DSX and 10 μg/mL pyoverdine (PA14). (D) Δ*fpvA*, Δ*fpvA* Δ*fpvB*, and Δ*pvdA* Δ*pchA* strain growth. Blue indicates control, purple indicates treatment with 64 μg/mL DSX, grey indicates treatment with 10 μg/mL ferrichrome, and red indicates treatment with 64 μg/mL DSX and 10 μg/mL ferrichrome. (E) Δ*fpvA*, Δ*fpvA* Δ*fpvB*, and Δ*pvdA* Δ*pchA* strain growth. Blue indicates control, purple indicates treatment with 64 μg/mL DSX, grey indicates treatment with 10 μg/mL ferrioxamine B, and red indicates treatment with 64 μg/mL DSX and 10 μg/mL ferrioxamine B. Results are averaged from three independent biological replicates. Download FIG S1, EPS file, 8.5 MB. Copyright © 2022 Chan and Burrows. 2022 Chan and Burrows. https://creativecommons.org/licenses/by/4.0/ This content is distributed under the terms of the Creative Commons Attribution 4.0 International license.

Another explanation for the susceptibility of the Δ*fpvA* mutant to DSX is that the mutant produces less pyoverdine compared to WT cells due to loss of the signaling cascade that controls siderophore production in response to its binding to FpvA (25). To differentiate whether the *fpvA* mutant was susceptible to DSX because of reduced pyoverdine production or because FpvB was a poor pyoverdine transporter, we treated the WT, Δ*fpvA*, Δ*fpvB*, and Δ*fpvA* Δ*fpvB* strains with DSX (64 μg/mL) without and with exogenous pyoverdine (10 μg/mL) for 20 h (Fig. 1; see also Fig. S1C). Growth of the Δ*fpvA* mutant should be restored if there are functional pyoverdine transporters. For WT and the Δ*fpvB* mutant, growth was similar between the control and DSX conditions. Pyoverdine or DSX plus pyoverdine treatment increased growth. For the Δ*fpvA* mutant, DSX inhibited growth and pyoverdine supplementation delayed growth. DSX plus pyoverdine treatment restored growth compared to DSX alone but also further delayed growth compared to the pyoverdine-alone treatment. The growth of the Δ*fpvA* Δ*fpvB* mutant was inhibited by DSX and pyoverdine, confirming previously published results (18). Taken together, this suggests that the FpvB is a less efficient pyoverdine transporter than FpvA.

As a control, a Δ*pvdA* Δ*pchA* mutant unable to make pyoverdine and pyochelin was also tested. Growth of this mutant was inhibited by DSX. We showed previously that a *pvdA* mutant remains susceptible to TS, indicating that it makes functional pyoverdine transporters even though the signaling cascade controlled by FpvA is disrupted in the absence of ligand production (13). As expected, pyoverdine restored growth of the mutant in the presence of DSX (Fig. S1C). As additional controls, the Δ*fpvA*, Δ*fpvA* Δ*fpvB*, and Δ*pvdA* Δ*pchA* mutants were treated with DSX in the presence of the xenosiderophores ferrichrome and ferrioxamine B, which use FiuA and FoxA for uptake (Fig. S1D and E) (17). The two xenosiderophores rescued growth of all three mutants in the presence of DSX within a 20-h incubation period, without the delay in growth seen with pyoverdine. Taken together, these data suggest that FpvB is a poor pyoverdine transporter.

The Δ*fpvA* Δ*fpvB* mutant was complemented in *trans* with pHERD20T-*fpvA* or pHERD20T-*fpvB*. pHERD20T is an arabinose-inducible vector with expression driven by the P_BAD_ promoter under the control of AraC (30). Complementation with *fpvA* or *fpvB* and induction with arabinose restored TS susceptibility, although susceptibility was greater with *fpvB* (Fig. 2C). However, only complementation with *fpvA* restored growth with DSX (Fig. 2D). Since *fpvB* could not restore growth of the double mutant in the presence of DSX, we hypothesized that FpvB is a poor transporter of pyoverdine but may transport other siderophores.

### FpvB is a transporter for ferrichrome and ferrioxamine B.

We investigated this hypothesis by treating the Δ*fpvA* Δ*fpvB* mutant complemented with *fpvA* or *fpvB* with TS and pyoverdine-Fe^3+^. If pyoverdine competes with TS for the same binding site in the transporter, a reduction in TS susceptibility would be expected, as competition would decrease entry of the antibiotic into the cell. TS susceptibility decreased >8-fold with increasing concentrations of pyoverdine-Fe^3+^ when FpvA was expressed (see Fig. S2A in the supplemental material). However, pyoverdine-Fe^3+^ did not impact TS susceptibility when FpvB was expressed under the same conditions (Fig. 2E). These results show that pyoverdine is a poor competitor for FpvB binding. As controls, we tested TS with ferrichrome, ferrioxamine B, enterobactin, ferrioxamine E, and arthrobactin, siderophores not expected to use FpvA or FpvB for uptake (Fig. S2B through F). None of those siderophores reduced TS susceptibility when FpvA was expressed, showing that only pyoverdine competes with TS for FpvA binding.

10.1128/mbio.03149-22.2 FIG S2 Checkerboard assays of the PA14 Δ*fpvA* Δ*fpvB* strain complemented with *fpvA* and *fpvB in trans* with TS and siderophores. Checkerboards of the PA14 Δ*fpvA* Δ*fpvB* pHERD20T-*fpvA* strain with TS and pyoverdine-Fe^3+^ (A), ferrichrome (B), ferrioxamine B (C), enterobactin (D), ferrioxamine E (E), and arthrobactin (F). Checkerboards of the PA14 Δ*fpvA* Δ*fpvB* pHERD20T-*fpvB* strain with TS and enterobactin (G), ferrioxamine E (H), and arthrobactin (I). Results are averaged from three independent biological replicates. Cells in the assay were grown in 10:90 + 2% arabinose. Download FIG S2, EPS file, 2.2 MB. Copyright © 2022 Chan and Burrows. 2022 Chan and Burrows. https://creativecommons.org/licenses/by/4.0/ This content is distributed under the terms of the Creative Commons Attribution 4.0 International license.

Surprisingly, ferrichrome and ferrioxamine B, but not other siderophores, antagonized TS susceptibility when FpvB was expressed (Fig. 2F and G; see also Fig. S2G through I). These data suggested that FpvB may transport these two xenosiderophores. We generated 15 single, double, triple, and quadruple knockout mutants lacking different combinations of *fpvA*, *fpvB*, *foxA*, and *fiuA* and assessed their growth in the presence of 64 μg/mL DSX, 10 μg/mL ferrichrome, 10 μg/mL ferrioxamine B, and 10 μg/mL ferrioxamine E to determine ligand specificity (Fig. 2H). If there were no transporters for a particular siderophore, the mutant would fail to grow due to iron restriction. TS was used as a control at 17 μg/mL since Δ*fpvB* and Δ*fpvA* Δ*fpvB* mutants are resistant to the antibiotic (Fig. 2A). Δ*fpvA* mutants were unable to grow in the presence of DSX, consistent with previous results (Fig. 1B; see also Fig. S1C). Growth of any mutant combination that included the Δ*foxA* mutation was inhibited by ferrioxamine E, confirming previous work showing that ferrioxamine E exclusively uses FoxA as a transporter in P. aeruginosa (27). Δ*fpvB* Δ*foxA* mutants failed to grow in the presence of ferrioxamine B, while Δ*fpvB* Δ*fiuA* mutants failed to grow with ferrichrome. The Δ*fpvA* Δ*fpvB* Δ*fiuA* Δ*foxA* quadruple mutant failed to grow with DSX, ferrioxamine B, ferrioxamine E, or ferrichrome and was resistant to TS. These results suggest that ferrichrome can be taken up via FpvB and FiuA, whereas ferrioxamine B can be taken up via FpvB and FoxA. The siderophores and chelators were bacteriostatic rather than bactericidal, as serial dilution onto nonselective media of PA14 and the Δ*fpvA* Δ*fpvB* Δ*fiuA* Δ*foxA* mutant after treatment with the different compounds resulted in regrowth (Fig. 2I and J).

### FpvB has higher affinity for ferrichrome and ferrioxamine B than pyoverdine.

**FIG 3:**
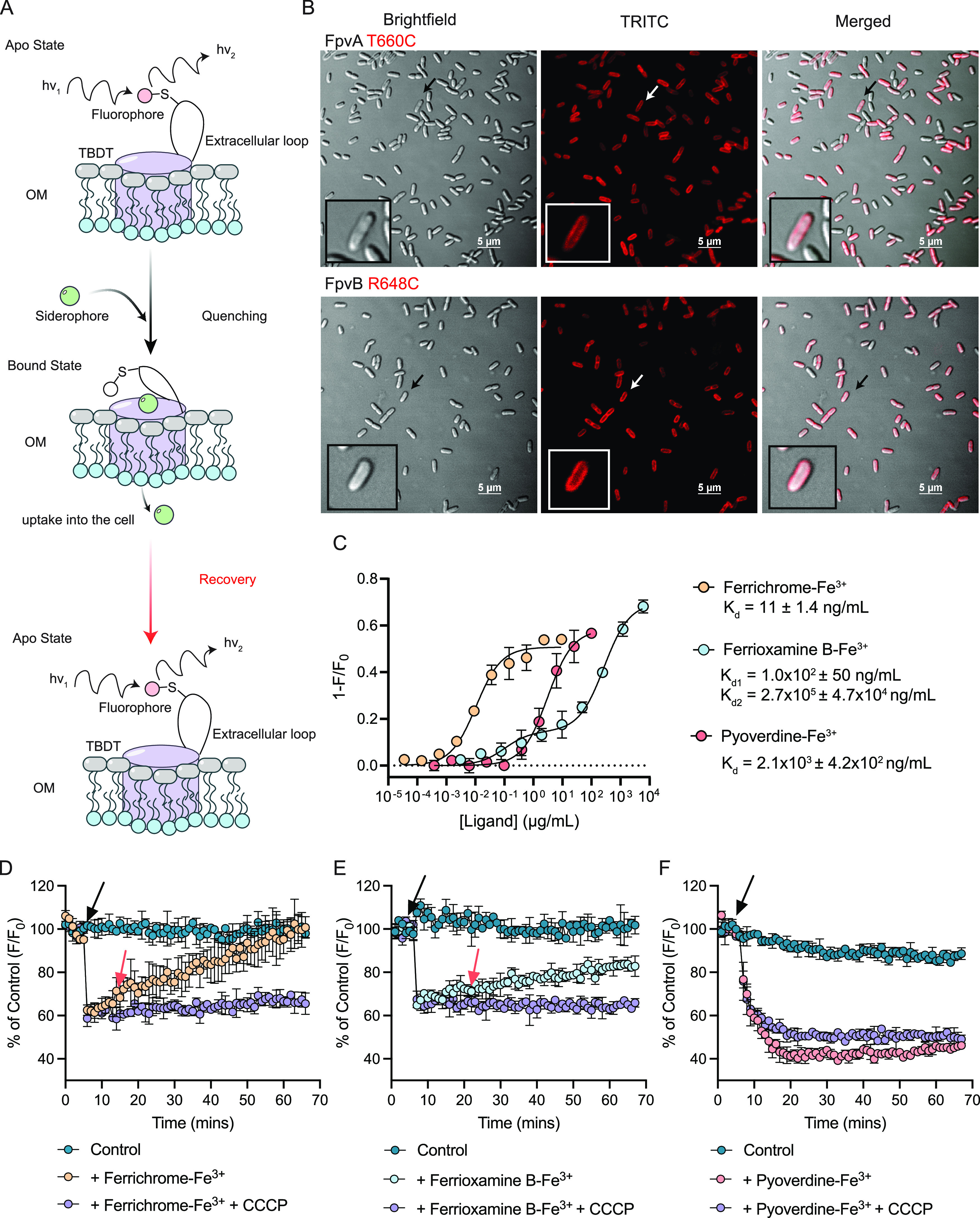
FpvB interacts with ferrichrome, ferrioxamine B, and pyoverdine. (A) Schematic for fluorescence quenching of site-directed labeling of TBDT Cys mutants. A Cys residue is introduced in the extracellular loops of a TBDT of interest and labeled with maleimide dye. A siderophore recognized by the TBDT binds the transporter, inducing conformational changes at the labeled extracellular loop. Changes in the chemical environment surrounding the fluorophore quenches fluorescence. Uptake of the siderophore into the cell restores fluorescence as the loop returns to its original conformation. (B) Fluorescence microscopy images of PA14 Δ*fpvA* Δ*fpvB* pHERD20T-*fpvA* T660C and *fpvB* R684C strains labeled with Alexa Fluor 594. (Inset) Zoomed in view of a single labeled cell. Scale bar is 5 μm. (C) Fluorescence quenching of labeled FpvB R648C by ferrichrome-Fe^3+^ (blue circles), ferrioxamine B-Fe^3+^ (red circles), or pyoverdine-Fe^3+^ (orange circles). *K_d_* values are shown for ferrichrome-Fe^3+^ and pyoverdine-Fe^3+^. Fluorescence recovery of labeled FpvB R648C at 1/2 *K_d_* for ferrichrome-Fe^3+^ (orange circles) (D), ferrioxamine B-Fe^3+^ (blue circles) (E), and pyoverdine-Fe^3+^ (red circles) (F). Teal circles represent vehicle controls, and purple circles represent siderophores + 20 μM CCCP. The black arrow indicates when each siderophore complex was added. The red arrow highlights when recovery is observed. No recovery was observed at any pyoverdine-Fe^3+^ concentration. All results are averaged from three independent biological replicates except for the microscopy images where a representative image is shown.

To determine the affinity of the siderophores for FpvA and FpvB, we adapted the method from Chakravorty et al. to generate a whole-cell sensor where fluorescence quenching serves as an indicator of ligand interaction (31). Siderophore binding triggers conformational changes at the extracellular loops of the TBDT (Fig. 3A). Cys substitutions were introduced at the loops and labeled with a fluorescent maleimide dye. When a ligand binds the transporter, the loops fold inward toward the lumen of the barrel, and changes in the chemical environment surrounding the fluorophore lead to quenching (31, 32). Fluorescence recovery occurs once the siderophore is taken up and the loop returns to its original conformation.

FpvA T660C was used previously to measure pyoverdine-Fe^3+^ binding affinity (32). However, this technique has not been applied to FpvB. A high-confidence structural model of FpvB was generated using AlphaFold2 (33, 34) and aligned with the structure of FpvA (PDB accession number 2O5P) to identify a residue suitable for labeling, with the assumption that FpvA and FpvB undergo similar conformational changes upon ferrisiderophore binding (see Fig. S3A through C in the supplemental material). Based on this analysis, FpvB R648 was mutated to Cys. FpvA T660C and FpvB R648C were each expressed in *trans* in the Δ*fpvA* Δ*fpvB* mutant. FpvB R648C had WT susceptibility to TS and growth in the presence of DSX (Fig. S3D). Expression of FpvA T660C increased susceptibility to TS by 4-fold compared to WT FpvA and restored growth with DSX. We could also detect expression by fluorescence microscopy with peripheral labeling consistent with expression in the outer membrane (Fig. 3B). Labeling was undetectable in empty vector and WT controls (see Fig. S4A in the supplemental material).

10.1128/mbio.03149-22.3 FIG S3 Overlay of FpvA and FpvB showing residues mutated to cysteine for labeling with maleimide dyes. FpvA (PDB accession number 2O5P) was superimposed with the AlphaFold2 model of FpvB. FpvA is shown in beige and FpvB in blue. Side view (A), top view (B), and zoomed in view (C) of the labeled extracellular loop 8. (D) FpvB R648C growth with TS or DSX is similar to WT. FpvA T660C increases susceptibility to TS by 4-fold and restores growth with DSX. Results are averaged from three independent biological replicates. Assays were conducted in 10:90 + 2% arabinose. Download FIG S3, EPS file, 8.7 MB. Copyright © 2022 Chan and Burrows. 2022 Chan and Burrows. https://creativecommons.org/licenses/by/4.0/ This content is distributed under the terms of the Creative Commons Attribution 4.0 International license.

10.1128/mbio.03149-22.4 FIG S4 (A) Microscopy images of the PA14 Δ*fpvA* Δ*fpvB* strain with pHERD20T, pHERD20T-*fpvA*, and pHERD20T-*fpvB* treated with Alexa Fluor 594. Representative images are shown. Scale, 5μm. (B) TS partly quenches fluorescence of fluorescein-5-maleimide in the absence of cells. Each compound at the highest concentration tested was added to 10μM of fluorescein-5-maleimide in PBS in triplicate. TS reduced fluorescence to about 80% of the control. (Excitation, 485nm; emission, 528nm). ***, *P* < 0.001 (Student’s *t* test). (C) Pyoverdine-Fe^3+^, ferrioxamine B-Fe^3+^, and ferrichrome-Fe^3+^ quenching curves with the PA14 Δ*fpvA* Δ*fpvB* pHERD20T-*fpvA* T660C strain. Results are averaged from three independent biological replicates. Download FIG S4, EPS file, 39.8 MB. Copyright © 2022 Chan and Burrows. 2022 Chan and Burrows. https://creativecommons.org/licenses/by/4.0/ This content is distributed under the terms of the Creative Commons Attribution 4.0 International license.

Ferrichrome-Fe^3+^, ferrioxamine B-Fe^3+^, and pyoverdine-Fe^3+^ were titrated at increasing concentrations into cells expressing FpvA T660C and FpvB R648C labeled with fluorescein-5-maleimide. TS was omitted from these studies because it quenched fluorescence (~20%) of the dye in the absence of cells (Fig. S4B). In the absence of protein, none of the three siderophores quenched fluorescence of the dye at the highest concentrations tested. Quenching curves were then generated and used to calculate the dissociation constant (*K_d_*). Pyoverdine-Fe^3+^ strongly quenched fluorescence of cells expressing FpvA T660C, with a *K_d_* of 10 ± 1.6 ng/mL (8.2 ± 1.2 nM), similar to values reported in previous studies (Fig. S4C) (32, 35). Ferrichrome-Fe^3+^ weakly quenched the fluorescence of cells expressing FpvA T660C (~20%), suggesting that ferrichrome may interact weakly with FpvA. No antagonism was observed between TS and ferrichrome, suggesting that the competition is minimal. No quenching was observed with ferrioxamine B-Fe^3+^.

The estimated *K_d_* of pyoverdine-Fe^3+^ for FpvB was 200-fold higher than FpvA (2.1 × 10^3^ ± 4.2 × 10^2^ ng/mL or 1.7 × 10^3^ ± 3.4 × 10^2^ nM), confirming that FpvA has higher affinity for pyoverdine (Fig. 3C). Ferrichrome-Fe^3+^ and ferrioxamine B-Fe^3+^ also quenched the fluorescence of cells expressing FpvB R648C (Fig. 3C). The *K_d_* of ferrichrome-Fe^3+^ for FpvB was 11 ± 1.4 ng/mL (15 ± 1.9 nM). Titration of ferrioxamine B-Fe^3+^ yielded a curve with two quenching events, suggesting that it may bind at two sites on FpvB—one of higher affinity than the other. For *K_d_*_1_, the binding constant was 1.0 × 10^2^ ± 50 ng/mL and *K_d_*_2_ was 2.7 × 10^5^ ± 4.7 × 10^4^ ng/mL. These results may explain the pattern of antagonism between the siderophores and TS. Ferrichrome, which has the highest affinity for FpvB, also had the greatest impact on TS susceptibility. The *K_d_*_1_ of ferrioxamine B is approximately 10-fold greater than ferrichrome, suggesting reduced affinity for FpvB but was sufficient to antagonize TS susceptibility. However, the *K_d_* of pyoverdine for FpvB is 200- and 20-fold greater than ferrichrome and ferrioxamine B, respectively, and it had the least impact on TS susceptibility.

Siderophore uptake by FpvB was monitored by fluorescence recovery at a concentration of 1/2 *K_d_*. In the case of ferrioxamine B, we chose 1/2 *K_d_*_2_ since the quenching signal was greater. Cells were equilibrated for 5 min prior to introduction of the siderophores. Fluorescence was recorded every min for 1 h. Carbonyl cyanide *m*-chlorophenylhydrazone (CCCP) was used as a control to inhibit fluorescence recovery through dissipation of the proton motive force (PMF) (2, 3), which is required for uptake. Full fluorescence recovery was seen with ferrichrome (Fig. 3D). For ferrioxamine B, fluorescence recovered to ~50% of the control; however, no recovery was seen with pyoverdine, suggesting that uptake of this siderophore is slow (Fig. 3E and F). These results suggest that of the three siderophores, ferrichrome has the greatest affinity for FpvB and is taken up most rapidly, followed by ferrioxamine B.

### Molecular determinants of TS, ferrichrome, and ferrioxamine B uptake through FpvB.

**FIG 4:**
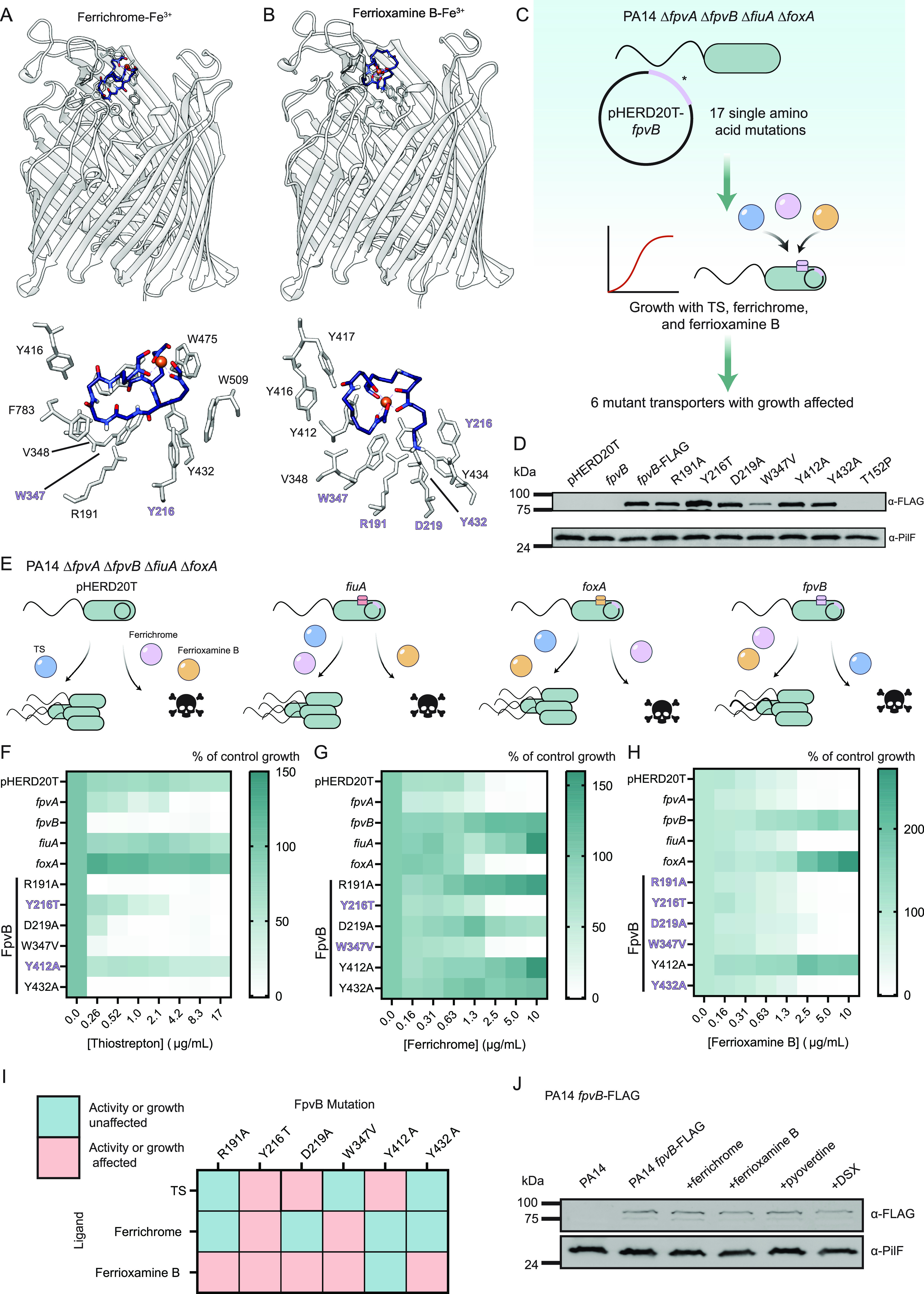
Molecular determinants of TS, ferrichrome, and ferrioxamine B uptake through FpvB. (A) Ferrichrome-Fe^3+^ (blue) (PDB accession number 1BY5) was docked into the AlphaFold2 model of FpvB (gray) using Autodock Vina. Predicted molecular interactions are shown below with residues important for uptake highlighted in purple and bolded. (B) Ferrioxamine B-Fe^3+^ (blue) (PDB accession number 6I96) was docked into the AlphaFold2 predicted model of FpvB (gray). Predicted molecular interactions are shown below with residues important for uptake highlighted in purple and bolded. (C) Schematic for validating the docking predictions. (D) Western blot of FLAG-tagged mutant FpvB transporters, with PilF as a loading control. Cells were grown in 10:90 + 2% arabinose. (E) Expected phenotypes for the PA14 Δ*fpvA* Δ*fpvB* Δ*foxA* Δ*fiuA* strain harboring pHERD20T, pHERD20T-*fpvB*, pHERD20T-*fiuA*, and pHERD20T-*foxA* treated with TS, ferrichrome, and ferrioxamine B. The PA14 Δ*fpvA* Δ*fpvB* Δ*foxA* Δ*fiuA* strain complemented with empty vector, *fpvA*, *fpvB*, *fiuA*, *foxA*, and *fpvB* mutants treated with TS (F), ferrichrome (G), and ferrioxamine B (H). Growth (green) is expressed as percent of control. Results are averaged from three independent biological replicates. Cells were grown in 10:90 + 2% arabinose. (I) Summary of effects of the mutations on TS susceptibility and growth in the presence of ferrichrome and ferrioxamine B. Teal, growth is affected; coral, growth is unaffected. (J) Western blot for WT PA14 with chromosomally integrated C-terminal FLAG-tagged FpvB treated with 10 μg/mL ferrichrome, 10 μg/mL ferrioxamine B, 10 μg/mL pyoverdine, and 64 μg/mL DSX. Cells were grown in 10:90. PilF was used as a loading control for outer membrane proteins.

Our data suggested that because ferrichrome, ferrioxamine B, and pyoverdine all quenched fluorescence, they might induce similar conformational changes in FpvB. Ferrichrome-Fe^3+^ and ferrioxamine B-Fe^3+^ were docked into the model of FpvB using AutoDock VINA to identify possible molecular interactions (33, 36). Docking assumed that the siderophore-Fe^3+^ complexes are in similar orientations in their native transporter and in FpvB. Based on the predictions, ferrichrome, and ferrioxamine B bind in a highly aromatic pocket with several Trp and Tyr residues, between the plug and barrel domains (Fig. 4A and B).

Site-directed mutagenesis was used to confirm the docking predictions and to define the molecular determinants for ligand uptake through FpvB (Fig. 4C). We generated 17 single amino acid substitutions in FpvB and expressed each of them in *trans* in the quadruple Δ*fpvA* Δ*fpvB* Δ*fiuA* Δ*foxA* mutant. Their ability to complement growth of the mutant in the presence of ferrichrome and ferrioxamine B and to restore TS susceptibility was assessed. Of the 17 mutations, the following six affected TS susceptibility and growth with ferrichrome and ferrioxamine B: R191A (plug domain), Y216T (extracellular loop), D219A (plug domain), W347V (extracellular loop), Y412A (barrel domain), and Y432A (barrel domain). The mutant transporters were tagged with a C-terminal FLAG tag to evaluate expression levels. FLAG-tagged FpvB mutants had WT levels of expression except for Y216T and W347V, which were expressed at 140% and 25% of WT, respectively. These results suggest that except for W347V, differences in growth are not due to differences in expression (Fig. 4D). FpvB-FLAG is functional and restores TS activity, ferrichrome uptake, and ferrioxamine B uptake (see Fig. S5A in the supplemental material).

10.1128/mbio.03149-22.5 FIG S5 (A) PA14 Δ*fpvA* Δ*fpvB* Δ*fiuA* Δ*foxA* strain with empty vector (pHERD20T) and complemented with *fpvB* and *fpvB-*FLAG treated with TS, ferrichrome, and ferrioxamine B in 10:90 + 2% arabinose. Heat map depicts growth where green indicates growth and white indicates no growth. Results are averaged from three independent biological replicates. (B) Checkerboard assays in the quadruple transporter mutant background between TS and ferrichrome. Growth is expressed as percent of control. Checkerboards were conducted in 10:90 + 2% arabinose. Results are averaged from three independent biological replicates. Download FIG S5, EPS file, 2.2 MB. Copyright © 2022 Chan and Burrows. 2022 Chan and Burrows. https://creativecommons.org/licenses/by/4.0/ This content is distributed under the terms of the Creative Commons Attribution 4.0 International license.

The Δ*fpvA* Δ*fpvB* Δ*fiuA* Δ*foxA* mutant was complemented with WT *fpvA*, *fpvB*, *fiuA*, and *foxA* in *trans* as controls (Fig. 4E). The quadruple mutant with the empty vector is resistant to TS and its growth inhibited by ferrichrome and ferrioxamine B. Complementation with *fpvA* was predicted to restore susceptibility to TS but not growth with ferrichrome or ferrioxamine B, while complementation with *fpvB* was expected to restore susceptibility to TS and growth with ferrichrome and ferrioxamine B. Complementation with *fiuA* was expected to restore growth with ferrichrome but not ferrioxamine B, while cells remain resistant to TS. Finally, complementation with *foxA* was expected to restore growth with ferrioxamine B but not ferrichrome, while cells remain resistant to TS. Several mutations within the binding pocket negatively affected TS susceptibility or growth with ferrichrome and ferrioxamine B (Fig. 4F through H). Y216T, D219A, and Y412A reduced TS susceptibility. Y216T and W347V prevented growth in the presence of ferrichrome. All mutations except Y412A prevented growth in the presence of ferrioxamine B. The effects of the mutations on TS susceptibility and growth in the presence of ferrichrome and ferrioxamine B are summarized in Fig. 4I. Finally, we tested whether ferrichrome, ferrioxamine B, and pyoverdine could stimulate FpvB expression (Fig. 4J). DSX was included as a negative control since it does not bind FpvB. None of the compounds stimulated expression, consistent with previous proteomic and reverse transcriptase PCR (RT-PCR) assays (27).

**FIG 5:**
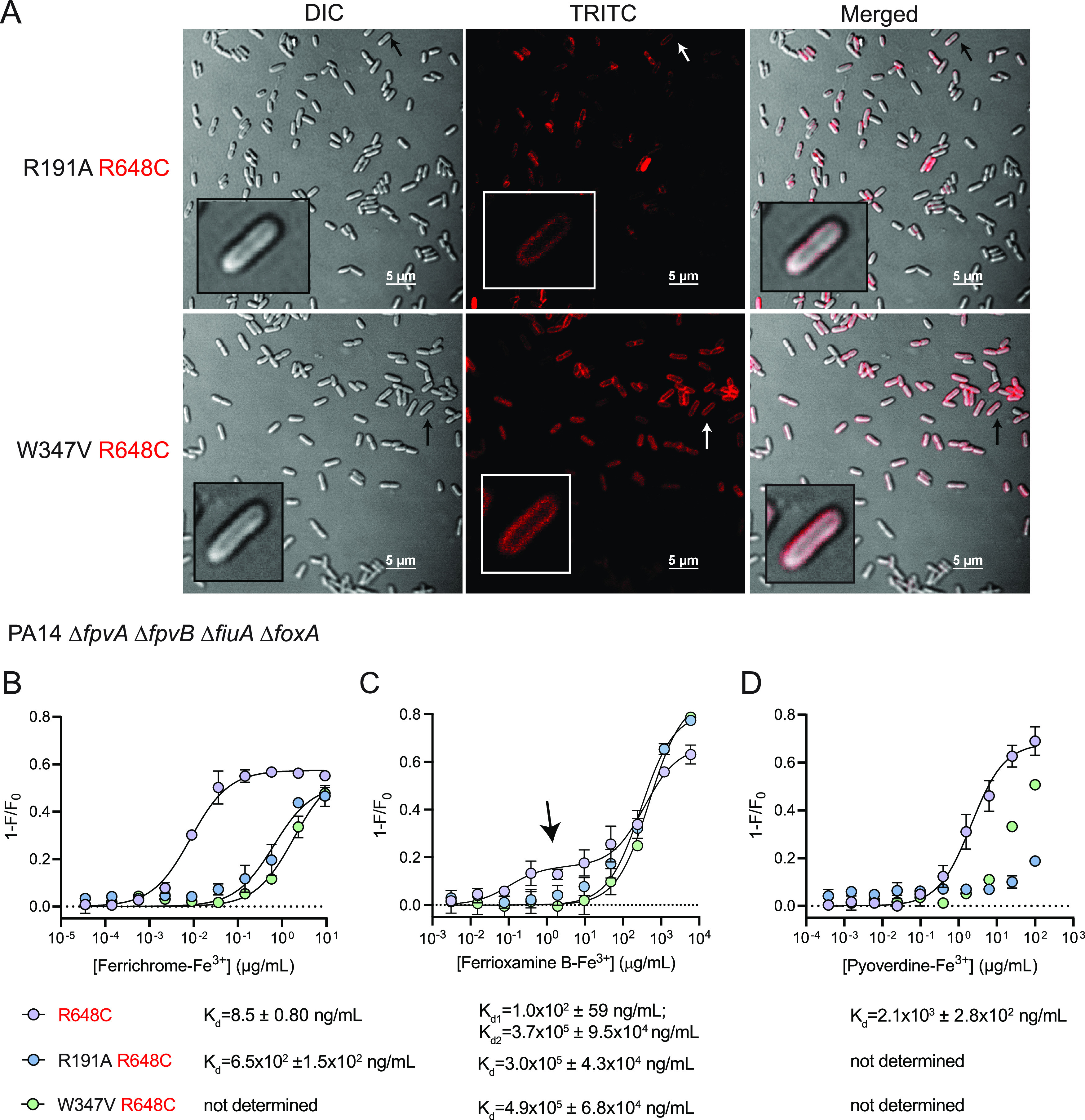
FpvB single-residue mutants are stably expressed but have reduced affinity for siderophores. (A) Microscope images of the Δ*fpvA* Δ*fpvB* Δ*fiuA* Δ*foxA* strain expressing FpvB R191A R648C or W347V R648C labeled with Alexa Fluor 594. Scale bar, 5 μm. Quenching curves of the PA14 Δ*fpvA* Δ*fpvB* Δ*fiuA* Δ*foxA* strain expressing fluorescein-5-maleimide-labeled FpvB R648C (purple), R191A R648C (blue), and W347V R648C (green) titrated with ferrichrome-Fe^3+^ (B), ferrioxamine B-Fe^3+^ (C), or pyoverdine-Fe^3+^ (D). The arrow in panel B highlights the first saturation event observed with FpvB R648C titrated with ferrioxamine B-Fe^3+^. Results are averaged from three independent biological replicates.

To determine if these mutations affected binding of ferrichrome and ferrioxamine B to FpvB, we introduced R648C to allow fluorescent labeling of the six point mutants; however, only R191A and W347V could be labeled with Alexa Fluor 594 (Fig. 5A). Quenching assays with the two xenosiderophores were repeated for the quadruple TBDT mutant expressing WT FpvB, FpvB R191A R648C, or W347V R648C. The R191A mutation increased the *K_d_* of ferrichrome-Fe^3+^ by 76-fold to 6.5 × 10^2^ ± 1.5 × 10^2^ ng/mL (8.7 × 10^2^ ± 2.0 × 10^2^ nM) while the W347V mutation increased the *K_d_* >76-fold (Fig. 5B). FpvB R191A allowed growth of the quadruple mutant in the presence of ferrichrome whereas W347V did not (Fig. 4G and H). These data suggest that certain mutations in FpvB can be tolerated and allow sufficient ferrichrome uptake, potentially because the *K_d_* of ferrichrome for WT FpvB is naturally low.

R191A and W347V also compromised ferrioxamine B uptake, suggesting reduced affinity (Fig. 4H and I). This was confirmed with the fluorescence quenching assay using the quadruple transporter mutant expressing FpvB R648C (Fig. 5C). The *K_d_*_1_ and *K_d_*_2_ were 1.0 × 10^2^ ± 59 ng/mL (1.7 × 10^2^ ± 96 nM) and 3.7 × 10^5^ ± 9.5 × 10^4^ ng/mL (6.0 × 10^5^ ± 1.5 × 10^4^ nM), similar to the Δ*fpvA* Δ*fpvB* mutant (Fig. 3C and Fig. 5C). The quenching curves for the R191A and W347V mutants fit a typical one-site model rather than the two-site model observed for the WT with *K_d_* values of 3.0 × 10^5^ ± 4.3 × 10^4^ ng/mL (4.8 × 10^5^ ± 7.0 × 10^4^ nM) and 4.9 × 10^5^ ± 6.8 × 10^4^ ng/mL (8.0 × 10^2^ ± 1.1 × 10^2^ nM), respectively. Neither mutation affected the *K_d_*_2_. These results suggest that mutating the predicted binding pocket prevents the initial interaction of ferrioxamine B with FpvB and further supports the hypothesis that ferrioxamine B binds at two distinct sites. Additionally, conformational changes that occur from the first binding event appear to be independent from the second since the quenching is still observed in the mutants.

Pyoverdine-Fe^3+^ binding to FpvB single residue mutants was also assessed using the fluorescence quenching assay. The *K_d_*s of pyoverdine for FpvB were similar in the Δ*fpvA* Δ*fpvB* mutant and the quadruple mutant (Fig. 3C and Fig. 5D). For the R191A and W347V mutants, the *K_d_* could not be determined because saturation was not reached even at the highest concentration tested, indicating that pyoverdine has reduced affinity for the two FpvB mutants. Overall, the R191A and W347V mutations reduced the affinity of FpvB for all three ligands.

Since only a subset of FpvB mutant transporters could be fluorescently labeled, we tested competition between TS and ferrichrome via checkerboard assays. The quadruple transporter mutant complemented with WT FpvB or FpvB single-residue mutants was treated with ferrichrome and TS and assessed for antagonism of TS susceptibility (Fig. S5B). Ferrioxamine B was not tested because the quadruple mutant expressing 5 of 6 FpvB single residue mutations was unable to grow with that siderophore (Fig. 4H and I). Antagonism was observed between ferrichrome and TS when WT FpvB was expressed. Consistent with the data in Fig. 4G, the Y216T and W347V mutant FpvB transporters were unable to support growth with ferrichrome. Interestingly, while R191A and D219A supported growth of the quadruple mutant in the presence of ferrichrome, no competition with TS was observed, suggesting that these mutations specifically reduce the affinity of FpvB for ferrichrome. When the Y432A mutant transporter was expressed, ferrichrome antagonized TS activity, although to a lesser extent than that observed with the WT. Overall, the mutations diminished the ability of ferrichrome to compete with TS.

To identify any other proteins important for susceptibility to TS, we raised mutants resistant to TS plus DSX. PA14 was grown in liquid cultures with 17 μg/mL of TS plus 64 μg/mL DSX and passaged over a period of 3 weeks. Single colonies were isolated on agar containing 17 μg/mL of TS plus 64 μg/mL DSX, and 6 were selected for sequencing. Two mutations that conferred resistance were identified. All resistant mutants had a premature stop codon in *tonB1* after residue A36, suggesting that functional TonB1 is not made. We showed previously that *tonB1* mutants are resistant to thiopeptides (14). A mutation in *fpvB* (T152P) was also identified in two colonies. FpvB T152P was tagged with a C-terminal FLAG tag and expressed in *trans* to evaluate expression, but no detectable bands were present from outer membrane preparations (Fig. 4D). Therefore, this mutation likely abolishes expression of functional FpvB. No mutations in FpvA were identified, although this may be due to selective pressure from DSX since we showed that FpvA is important for growth with the chelator (Fig. 2B and D).

## DISCUSSION

This work expands on the discovery of FpvB as an alternative transporter for pyoverdine (18). Ghysels et al. (18) made a P. aeruginosa PAO1 mutant unable to produce FpvA, pyoverdine, or pyochelin, which grew in Casamino Acids (CAA) medium. Supplementing the media with ethylene diamine di(ο-hydroxyphenyl)acetic acid (EDDHA), an iron chelator unable to enter cells, inhibited growth, similar to our results with the PA14 Δ*fpvA* mutant and DSX (see Fig. S1C in the supplemental material). Supplementing the media with pyoverdine restored growth of the mutant after 24 h but not 12 h, suggesting expression of a TBDT with limited affinity for pyoverdine. Deleting *fpvB* inhibited growth recovery, suggesting that FpvB transports pyoverdine. Another study showed that a PAO1 Δ*fpvA* mutant was unable to grow in media supplemented with EDDHA even if pyoverdine was added, although growth was only monitored for 10 h rather than 24 h (37). Similarly, a PAO1 FpvA-deficient strain was unable to take up iron from pyoverdine-^59^Fe, regardless of whether pyoverdine from PAO1 or other pseudomonads was provided, although uptake was measured only for 15 min (38). Together, these results suggest that FpvB is a poor transporter for pyoverdine, which informed our hypothesis that FpvB may transport other siderophores.

**FIG 6:**
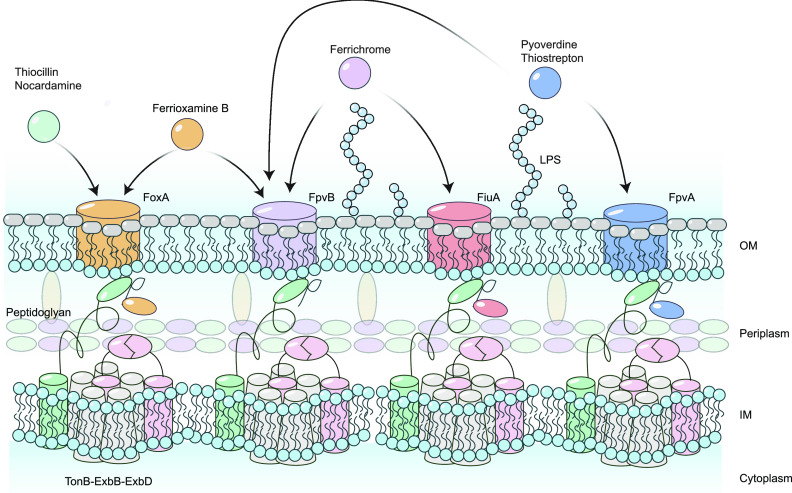
Model for thiopeptide and siderophore uptake through the TBDTs FpvA, FpvB, FoxA, and FiuA in P. aeruginosa. OM, outer membrane; IM, inner membrane.

In this work, we uncovered additional uptake pathways for the fungal siderophore, ferrichrome, and the bacterial siderophore, ferrioxamine B, in P. aeruginosa. Canonically, ferrichrome is taken up via FiuA while ferrioxamine B uses FoxA. Both siderophores can be recognized by FpvB (Fig. 6), and ferrichrome binds with greater affinity than ferrioxamine B or pyoverdine based on fluorescence quenching data (Fig. 3C). Unexpectedly, the quenching curve of ferrioxamine B-Fe^3+^ supported a two-site binding model. This result suggests that the binding mode of ferrioxamine B for FpvB is different than those of ferrichrome and pyoverdine and shows that different ligands can interact in distinct ways with the same TBDT. A two-site model has been proposed for the binding of enterobactin to its transporter PfeA, but ferrioxamine B and E were reported to bind their primary transporter FoxA only at a single site (4, 5, 27). We also used fluorescence recovery as an indicator of ligand uptake. Fluorescence recovery was not observed for pyoverdine even after 1 h (Fig. 3F), suggesting that pyoverdine uptake through FpvB is slow. A combination of a high *K_d_* and slow uptake may explain why FpvB is a poor transporter for pyoverdine.

The site-directed mutagenesis data suggest that all three ligands bind a similar hydrophobic pocket in FpvB, but the molecular determinants for uptake depend on the ligand. For example, W347 is important for both ferrichrome and ferrioxamine B but not TS uptake. Y412 is important for TS uptake but not for the two xenosiderophores, and Y216 is essential for uptake of all three ligands. Costructures of FpvB with each of the ligands will be informative. Combining the site-directed mutagenesis data with fluorescence quenching and checkerboard competition assays, we showed that modifying the hydrophobic pocket of FpvB reduced its affinity for the ligands. With ferrioxamine B, the R191A and W347V mutations abolished the initial quenching event observed in WT FpvB (Fig. 5C). However, the second quenching event was unaffected, suggesting that the two binding events occur independently and that conformational changes caused by the first event are not required for the second. Future work will focus on locating the second site to understand how ferrioxamine B interacts with FpvB. Additionally, this work supports the idea that the binding mechanism of different siderophores can vary even for the same transporter.

Determining the ligand specificity of TBDTs can be challenging. Traditionally, sequence alignments of transporters with known ligands and transporters with unknown ligands were used to make inferences about the function of the new transporter. This method works well for those that share high sequence similarity. For example, the aerobactin transporter in P. aeruginosa was discovered by comparing the sequences of Escherichia coli IutA and P. aeruginosa ChtA, which share 46% identity (63% similarity) (39). FpvB was initially discovered as a secondary ferripyoverdine transporter using this method, as it shares 54% amino acid similarity with FpvA (18). However, alignments do not provide a comprehensive picture of the range of ligands that can be taken up. FpvB shares only 30 to 40% similarity with FoxA and FiuA, even though our data suggest that it has higher affinity for ferrichrome than pyoverdine.

Proteomic and RT-PCR approaches have also been used to identify the transporters for ferrioxamine E, ferrioxamine B, and ferrichrome in P. aeruginosa (27). FoxA expression was upregulated in the presence of ferrioxamine E and ferrioxamine B, while FiuA expression was upregulated by ferrichrome. However, only a subset of TBDTs have N-terminal signaling domains that respond to the presence of ferrisiderophores in a feed-forward regulation loop to increase their expression. Further, many TBDTs have redundant functions, and knocking out single transporters may be insufficient to abolish uptake, making it difficult to understand the complete uptake pathway for a ligand. As an alternative, competition between an antimicrobial and siderophore has been used to show that an antimicrobial exploits a particular TBDT for uptake (40, 41). Here, we used competition between TS and various siderophores to show that FpvB recognizes ferrichrome and ferrioxamine B, antagonizing TS susceptibility. This method may be applicable to study the range of ligands that can be recognized by TBDTs in bacteria besides P. aeruginosa. The disadvantage of this method is that an antimicrobial known to use the TBDT of interest for uptake is required.

Previous studies established that 93% of P. aeruginosa clinical and environmental isolates have *fpvB* (13, 18, 42). It may be advantageous to produce a single transporter that recognizes at least three different siderophores; therefore, the observation that 7% of isolates lack *fpvB* seems paradoxical. Loss of *fpvB* may improve the fitness of P. aeruginosa in the lungs of cystic fibrosis patients through genome reduction (43). Supporting this finding, we previously identified a TS-resistant but thiocillin-sensitive clinical isolate, C0379, missing ~800 bp from the 5′ region of *fpvB*, suggesting that it may have once produced functional FpvB (13). Δ*fpvB* mutants are also fitter than WT cells when treated with the antibiotic gentamicin (44). Its absence may reduce the metabolic burden on cells living in stressful environments whether due to nutrient limitation or antibiotic stress. However, the proportion of isolates from environmental or clinical sources lacking *fpvB* is similar, suggesting that there are multiple factors involved (42). For example, while we identified TS plus DSX-resistant mutants with a single point mutation in *fpvB* (T152P) that abolished its expression, we also identified resistant P. aeruginosa unable to make TonB1. Although not all P. aeruginosa clinical isolates produce FpvB, we previously tested 96 clinical isolates and found that 94/96 were susceptible to a combination of TS and DSX, suggesting that the majority of the isolates produce FpvB (28). Alignment of FpvB sequences from 32 of 96 clinical isolates revealed 99.8% amino acid identity, showing that it is highly conserved (see Fig. S6 in the supplemental material). These data support the idea that FpvB could be a clinically relevant uptake pathway for siderophore-antibiotic conjugates to treat P. aeruginosa infections.

10.1128/mbio.03149-22.6 FIG S6 Amino acid sequence alignments of FpvB in P. aeruginosa PA14 and 32 clinical isolates. White indicates an identical amino acid position across all 33 sequences. Black indicates a different amino acid. Download FIG S6, EPS file, 21.9 MB. Copyright © 2022 Chan and Burrows. 2022 Chan and Burrows. https://creativecommons.org/licenses/by/4.0/ This content is distributed under the terms of the Creative Commons Attribution 4.0 International license.

This work has implications for our understanding of how siderophores are taken up by P. aeruginosa compared to other bacteria. In E. coli, ferrichrome is recognized by FhuA whereas ferrioxamine B weakly binds FhuE, which does not take up ferrioxamine E or ferrichrome (45, 46). In P. aeruginosa, ferrichrome is recognized by FiuA and FpvB, ferrioxamine B is recognized by FoxA and FpvB, and ferrioxamine E is exclusively recognized by FoxA. FpvB is unusual in that TBDTs typically take up siderophore-iron complexes that are structurally related to their native siderophores, which suggests that FpvB may have a high degree of promiscuity compared to other TBDTs. These differences in uptake are important considerations in the design of broad-spectrum siderophore-antibiotic conjugates for Gram-negative pathogens.

There is growing interest in the use of antimicrobials that exploit TBDTs for uptake. For example, conjugating antibiotics to ferrichrome or ferrioxamine B may permit a compound to be taken up by both FpvB and FiuA or FoxA. The benefits of an antibiotic-siderophore conjugate that can use multiple receptors include a reduced chance of developing resistance, as cells would have to lose multiple TBDTs. In this context, it would be of interest to see how modifications to the siderophore structure affects binding and uptake. For example, one natural variation that prevents ferrioxamine E from using FpvB is that it is cyclic (Fig. 1), whereas ferrioxamine B is linear in its apo form; however, they both adopt similar cyclic conformations when bound to Fe^3+^. Further, ferrioxamine B has an amine tail predicted to interact with R191 that does not participate in iron chelation; this extension is absent in ferrioxamine E. Structure-activity relationship studies of ferrichrome and ferrioxamine B may further reveal ligand-transporter interactions to allow for the design of better siderophore-drug conjugates.

## MATERIALS AND METHODS

### Strains and primers.

All strains and primers used in this study are listed in the supplemental materials (see Table S1 in the supplemental material).

10.1128/mbio.03149-22.7 TABLE S1 Primers and strains used in this study. Download Table S1, XLSX file, 0.02 MB. Copyright © 2022 Chan and Burrows. 2022 Chan and Burrows. https://creativecommons.org/licenses/by/4.0/ This content is distributed under the terms of the Creative Commons Attribution 4.0 International license.

### Compounds and media.

Ferrichrome and fluorescein-5-maleimide were purchased from Cayman Chemicals. Ferrioxamine B was purchased from Calbiochem. Pyoverdine and pyoverdine-Fe^3+^ were purchased from Sigma. Arthrobactin was purchased from MolPort. Alexa Fluor 594 C_5_ maleimide was purchased from Fisher Scientific. Carbenicillin was purchased from AK Scientific. l-arabinose was purchased from BioShop. Glucose was purchased from Fisher Scientific. Stock solutions and powders were stored at −20°C. Lysogeny broth (LB) was purchased as a premixed powder from BioShop. 10:90 medium was prepared as previously described (13, 14). l-arabinose was prepared as a 20% (wt/vol) stock solution in 10:90 and filter sterilized (0.2-μm pore size; Fisherbrand).

### Molecular biology.

Chromosomal mutants were generated by allelic exchange using pEX18Gm (47). Primers flanking the upstream and downstream regions of each gene of interest were amplified from PA14 genomic DNA (Promega Wizard Genomic DNA purification kit) and extracted with GeneJet gel extraction kit (ThermoFisher). The upstream and downstream regions were joined by overlap extension PCR or ligation, digested with the indicated enzymes (FastDigest; ThermoFisher), and ligated into pEX18Gm to make each deletion construct (T4 DNA ligase; ThermoFisher) (47). The ligation mixtures were transformed into competent E. coli DH5α by heat shock with a recovery period of 2 to 3 h in LB. Cells were plated on LB 1.5% agar containing 15 μg/mL gentamicin supplemented with 5-bromo-4-chloro-3-indolyl-β-d-galactopyranoside (X-gal) for blue-white screening. The plates were incubated at 37°C overnight. Colony PCR was performed on white colonies to check for the correct inserts, and those with the insert were grown in LB plus 15 μg/mL gentamicin overnight at 37°C with shaking (200 rpm). Plasmids were isolated using GeneJet plasmid miniprep kit (ThermoFisher).

Plasmids with correct inserts were transformed into competent E. coli SM10 by heat shock with a recovery period of 2 to 3 h in LB. The cells were plated on LB 1.5% agar containing 10 μg/mL gentamicin and grown overnight at 37°C. One colony was picked and inoculated in LB plus 10 μg/mL gentamicin. The P. aeruginosa mutant of interest was also inoculated from a single colony in LB. Both cultures were grown overnight at 37°C with shaking (200 rpm). SM10 with the desired deletion construct was mated with PA14 by mixing equal volumes of each overnight culture in a 1.5-mL centrifuge tube. Cells were spun down, and the supernatant was removed. Cells were resuspended in 50 μL fresh LB, spotted on LB 1.5% agar, and incubated overnight at 37°C. Cells from the mating spot were streaked onto Pseudomonas isolation agar (PIA) (Difco) supplemented with 100 μg/mL gentamicin and incubated overnight at 37°C. Single colonies were streaked onto LB (no salt) plus 15% sucrose (BioShop) and incubated overnight at 37°C. To check for colonies with the correct deletion, 16 colonies were patched onto LB plus 15% sucrose and LB plus 30 μg/mL gentamicin and incubated overnight at 37°C. Patches that grew on the sucrose plates but not gentamicin plates were checked by colony PCR with primers flanking the deleted gene and internal primers and compared to WT controls. Patches with the desired deletions were streaked onto LB plus 15% sucrose to isolate single colonies, incubated overnight at 37°C and checked again by colony PCR. A single colony was inoculated into LB broth, and the process was repeated to generate double, triple, and quadruple mutants.

Complemented strains were made using the plasmid pHERD20T, an arabinose-inducible expression vector with the P_BAD_ promoter under the control of AraC (30). Primers flanking the gene of interest including the native ribosome binding site were amplified from P. aeruginosa PA14 genomic DNA, digested with the desired restriction enzymes, and ligated into pHERD20T digested with the same enzymes. Ligation mixtures were added to chemically competent DH5α and DNA introduced by heat shock with a recovery period of 1 to 2 h in LB at 37°C. All of the cells were plated on LB 1.5% agar supplemented 100 μg/mL ampicillin, X-gal, and 0.1% arabinose and incubated at 37°C overnight. White colonies were analyzed by colony PCR and colonies with plasmids containing the desired insert size were cultured in LB broth supplemented with 100 μg/mL ampicillin. Plasmids were isolated and inserts validated by restriction digest and electroporated into the desired P. aeruginosa strain or mutant with a recovery of 1 to 2 h in LB at 37°C. All of the cells were plated on LB 1.5% agar supplemented with 200 μg/mL carbenicillin (AK Scientific). A single colony was picked and grown in LB supplemented with 200 μg/mL carbenicillin overnight at 37°C and used to make glycerol stocks and for subsequent assays. Correct inserts were verified by Sanger sequencing by the McMaster Genomics Facility Mobix Lab.

### MIC assays.

MIC assays were conducted as previously described (13, 14). Overnight cultures were grown in LB from a glycerol stock at 37°C with shaking (200 rpm). Subcultures in 10:90 (1:100 dilution) were cultured for 4 h. Cells were adjusted to an optical density at 600 nm (OD_600_) of 0.1/500 in 10:90. All compounds were serially diluted 2-fold in dimethyl sulfoxide (DMSO) or H_2_O at 75× the final concentration. Plates were sealed to prevent evaporation and incubated at 37°C overnight in a shaking incubator (200 rpm). The next day, the OD_600_ was determined with a plate reader (Thermo Scientific) and normalized to percent of growth of the vehicle control after subtracting the OD_600_ from blank media.

### Checkerboard assays.

Checkerboards (8 rows by 8 columns) were conducted as previously described in a 96-well plate (Nunc) (13, 14). TS at 75× the final concentration dissolved in DMSO was added in columns from bottom to top in increasing concentrations. Siderophores at 75× the final concentration dissolved in DSMO were added from in rows from left to right in increasing concentrations. Four columns were used for vehicle controls (DMSO) and sterile controls. Media with bacteria as described in the MIC assays were added to obtain a final volume of 150 μL. Plates were sealed to prevent evaporation and incubated at 37°C overnight in a shaking incubator (200 rpm). The next day, the OD_600_ was determined with a plate reader (Thermo Scientific) and normalized to percent of growth of the vehicle control (DMSO) after subtracting the OD_600_ from blank media.

### Microscopy, fluorescence quenching, and recovery assay.

Cells were cultured overnight in LB with carbenicillin (200 μg/mL) at 37°C with shaking at 200 rpm. Cells were subcultured (1:200 dilution) in 10:90 plus 2% arabinose for 4 h without carbenicillin at 37°C with shaking at 200 rpm. Cells were harvested by centrifugation for 5 min and 6,000 × *g* and resuspended in sterile 1× phosphate-buffered saline (PBS) (pH 7.4); 1× PBS was made from a 10× stock (80 g NaCl, 2 g KCl, 26.8 g Na_2_ HPO_4_-7H_2_O, 2.4 g KH_2_PO_4_ in 1 L of deionized H_2_O) with 10 μM Alexa Fluor 594 C_5_ maleimide for microscopy or fluorescein-5-maleimide for fluorescence quenching and recovery assays and incubated in the dark at room temperature for 30 min on a shaking incubator at 37°C (200 rpm). Excess dye was quenched with 1 mM dithiothreitol (DTT) (Sigma) to stop the reaction. Cells were washed 3× with PBS. For fluorescence recovery assays, cells were incubated with 1× PBS plus 0.4% glucose at 37°C with shaking at 200 rpm. Glucose was prepared as a 20% stock (wt/vol) in 1× PBS and filter sterilized. Cells were spun down and washed 3× with 1× PBS and resuspended in 1× PBS for quenching assays and 1× PBS plus 0.4% glucose for fluorescence recovery assays.

For microscopy, Alexa Fluor 594 C_5_-maleimide-labeled cells were spotted onto a 1% agarose pad on a microscope slide. The agarose pad was mounted with a glass coverslip directly prior to imaging. Cells were imaged using brightfield and fluorescence microscopy on a Nikon A1 confocal microscope through a Plan Apo 60× (NA = 1.40) oil objective. Image acquisition was done using Nikon NIS Elements Advanced Research (Version 5.11.01 64 bit) software.

For fluorescence quenching and recovery assays, cells were diluted to an OD_600_ of 0.1 for both types of assays, and 148 μL was added into wells of a 96-well black plate (Corning). Fluorescence was recorded for 5 min at 1-min intervals at 37°C (BioTek Neo; excitation, 494 nm; emission, 520 nm). After 5 min, 2 μL of each serial dilution for ferrioxamine-Fe^3+^, ferrichrome-Fe^3+^, and pyoverdine-Fe^3+^ or DMSO for vehicle controls was added to each well. Fluorescence was recorded immediately for quenching assays and for 1 h at 1-min intervals for fluorescence recovery assays. Background fluorescence was subtracted from all fluorescence readings and 1-F/F_0_ was used to calculate the degree of quenching, where F_0_ is the initial fluorescence. *K_d_* was calculated using GraphPad Prism using a one-site specific binding model or a two-site model. For fluorescence recovery assays, background was subtracted from all fluorescence readings and F/F_0_ expressed as a percent of control was plotted versus time.

### Outer membrane preparations, SDS-PAGE, and Western Blots.

The quadruple mutant expressing mutant FpvB transporters was grown overnight in LB with 200 μg/mL carbenicillin at 37°C with shaking (200 rpm). Cells were subcultured 1:100 into 10:90 for 4 h and then diluted to an OD_600_ of 0.1/500 in 50 mL of fresh 10:90 plus 2% arabinose. Cultures were grown overnight at 37°C with shaking (200 rpm). Cells were harvested by centrifugation (5 min, 6,000 × *g*) and resuspended in 10 mM Tris, pH 8.0.

Cells were lysed by sonication (Misonix Sonicator 3000) on ice (30-s pulse, power level 5.0). Cell debris was removed by centrifugation (6,000 × *g*, 5 min, 4°C). Proteins were harvested at 21,000 × *g* for 30 min at 4°C. The pellet was resuspended in 100 μL deionized H_2_O and combined with 900 μL 11.1 mM Tris, 1% Sarkosyl (Fisher Scientific), pH 7.6, and incubated at room temperature for 30 min with shaking (200 rpm). Outer membrane pellets were collected by centrifugation at 21,000 × *g* for 30 min at 4°C.

Outer membrane preparations were resuspended in 20 μL 1× loading buffer for SDS-PAGE analysis. SDS-PAGE buffer was made from a 10× tris-glycine buffer stock (30.3 g tris, 144 g glycine, and 20 mL 10% SDS). Each lane was loaded with 10 μL of outer membrane prep and proteins separated at 80 V for 10 min and 120 V for 1.5 h. Proteins were transferred to nitrocellulose membranes (225 mA, 1 h) in transfer buffer (20% methanol, 100 mL of a 10× tris-glycine buffer stock without SDS) and blocked with 5% skim milk in PBS overnight at 4°C. Primary antibodies (mouse α-DYKDDDDK [Invitrogen; MA1-91878] and rabbit no. 3198 α-PilF) were used at 1:1,000 dilutions in PBS and incubated with the blot at room temperature for 1 h. Blots were washed 3× for 10 min with PBS and incubated with rabbit α-mouse-alkaline phosphatase for 2 h in PBS at 1:2,000 dilutions in PBS. Blots were washed with PBS 3× (10 min per wash) before incubation with alkaline phosphatase buffer (1 mM Tris, 100 mM NaCl, 5 mM MgCl_2_ pH 9.5) plus 5-bromo-4-chloro-3-indoyl phosphate (BCIP) plus nitro-blue tetrazolium (NBT) for 15 to 30 min. Blots were developed in the dark and imaged on an Azure C400 Imaging System. Band densities were quantified using ImageJ (48).

### Mutants resistant to TS.

PA14 was cultured in 10:90 supplemented with 17 μg/mL TS plus 64 μg/mL DSX at 37°C with shaking at 200 rpm (13). Cells were passaged when turbidity was evident (1:500 dilution) into fresh media with the same concentrations of TS plus DSX. This procedure was repeated for 3 weeks until cells grew overnight with TS plus DSX. The cells were streaked on to LB deferrated with FEC-1 to remove iron overnight at 37°C and supplemented with TS plus DSX. FEC-1 was supplied by Chelation Partners Inc. (Fe Pharmaceuticals) (49). Isolates were picked and cultured in LB, and chromosomal DNA was isolated and whole-genome sequencing performed by the McMaster Genomics Facility Mobix Lab. Breseq was used to identify mutations associated with TS plus DSX resistance (50).

### Structural comparisons and docking.

Structural models of FpvB were generated using AlphaFold2 (ColabFold) and compared to the crystal structure of FpvA (PDB accession number 2O5P) (33, 34). Structural comparisons of FpvB and FpvA were visualized using Chimera (51). Ferrichrome (PDB accession number 1BY5) and ferrioxamine B (PDB accession number 6I96) were docking into the AlphaFold2 model of FpvB using AutoDock VINA (36) with a box size of center_x = −7.729, center_y = 34.958, center_z = 113.797, size_x = 114, size_y = 116, and size_z = 118. The top pose generated was used for further studies.
